# Comparison of analysis approaches for multi-level vascular imaging data

**DOI:** 10.1186/1745-6215-16-S2-P237

**Published:** 2015-11-16

**Authors:** Julia Forman, Marie Fisk, Joseph Cheriyan, Simon Bond, Ian Wilkinson

**Affiliations:** 1 Cambridge Clinical Trials Unit, Cambridge University Hospitals, Cambridge, UK; 2 Division of Experimental Medicine and Immunotherapeutics, Cambridge University Hospitals, Cambridge, UK; 3 MRC Biostatistics Unit, Cambridge, UK

## 

Analysis methods for clinical trials with imaging endpoints are not well established. Here we will present and compare methods to analyse vascular inflammation as measured by ^18^F-FDG PET/CT imaging.

These methods have been developed for the EVOLUTION trial: An Evaluation of Losmapimod in patients with Chronic Obstructive Pulmonary Disease (COPD) with systemic inflammation stratified using fibrinogen (EudraCT Number 2011-004936-75), a randomised, double-blind, placebo-controlled trial. Patients were randomised to 7.5 mg losmapimod or matching placebo tablets twice daily for four months.

Vascular inflammation was assessed by ^18^F-FDG PET/CT scan at baseline and following treatment. Each scan covers up to six vessel segments per participant (left carotid, right carotid, and four aortic segments) and each segment is analysed in “slices”, generating a three-level data structure: participants, vessels, slices. For each slice, the mean and maximum inflammation level is recorded (as measured by the tissue-to-background ratio of the ^18^F-FDG tracer).

Here, we consider three analysis approaches to assess the treatment effect. 1: Data are pooled by vessel and by patient, by considering the change in the mean (or max) inflammation. 2: The most inflamed vessel segment in each patient is identified at baseline and followed-up in each patient. 3: A multi-level model incorporates the complete data set and data structure. Through comparing these analysis approaches, we aim to identify a method which provides an accurate measure of the treatment effect, and a straightforward interpretation.

